# Supplementary material for: Kidney-Specific Membrane-Bound Serine Proteases CAP1/Prss8 and CAP3/St14 Affect ENaC Subunit Abundances but Not Its Activity
Source: Cells. 2023 Sep 23;12(19):2342. doi: 10.3390/cells12192342 (PMC10572026; doi:10.3390/cells12192342)
Supplement: Supplementary file 1 [file cells-12-02342-s001.zip › cells-2579152-supplementary.pdf]

Supplementary figures

Kidney-Specific Membrane-Bound Serine Proteases CAP1/Prss8 and CAP3/St14 Affect ENaC Subunit Abundances but Not Its Activity

Elodie Ehret [1,2], Sévan Stroh [1], Muriel Auberson [1], Frédérique Ino [1,3], Yannick Jäger [1,4], Marc Maillard [5], Roman Szabo [6], Thomas H. Bugge [6], Simona Frateschi [1,7] and Edith Hummler [1,2,*]

1 Department of Biomedical Sciences, Faculty of Biology and Medicine, University of Lausanne, 1011 Lausanne, Switzerland; elodie.ehret@unil.ch (E.E.); sevan.stroh@me.com (S.S.); muriel.auberson@unil.ch (M.A.); frederique.ino@unige.ch (F.I.); yannick.jaeger@mpi-bn.mpg.de (Y.J.); simona.frateschi@epfl.ch (S.F.)
2 National Center of Competence in Research "Kidney.CH", 1011 Lausanne, Switzerland
3 Department of Medicine, University of Geneva, 1211 Geneva, Switzerland
4 Department of Pharmacology, Max-Planck-Institute for Heart and Lung Research, 61231 Bad Nauheim, Germany
5 Service of Nephrology, Department of Medicine, Lausanne University Hospital (CHUV), 1005 Lausanne, Switzerland; marc.maillard@chuv.ch
6 National Institutes of Health/NIDCR, Bethesda, MD 20892, USA; rszabo@nidcr.nih.gov (R.S.); tbugge@dir.nidcr.nih.gov (T.H.B.)
7 Present address: Ecole Polytechnique Fédéral Lausanne (EPFL), 1011 Lausanne, Switzerland
* Correspondence: edith.hummler@unil.ch

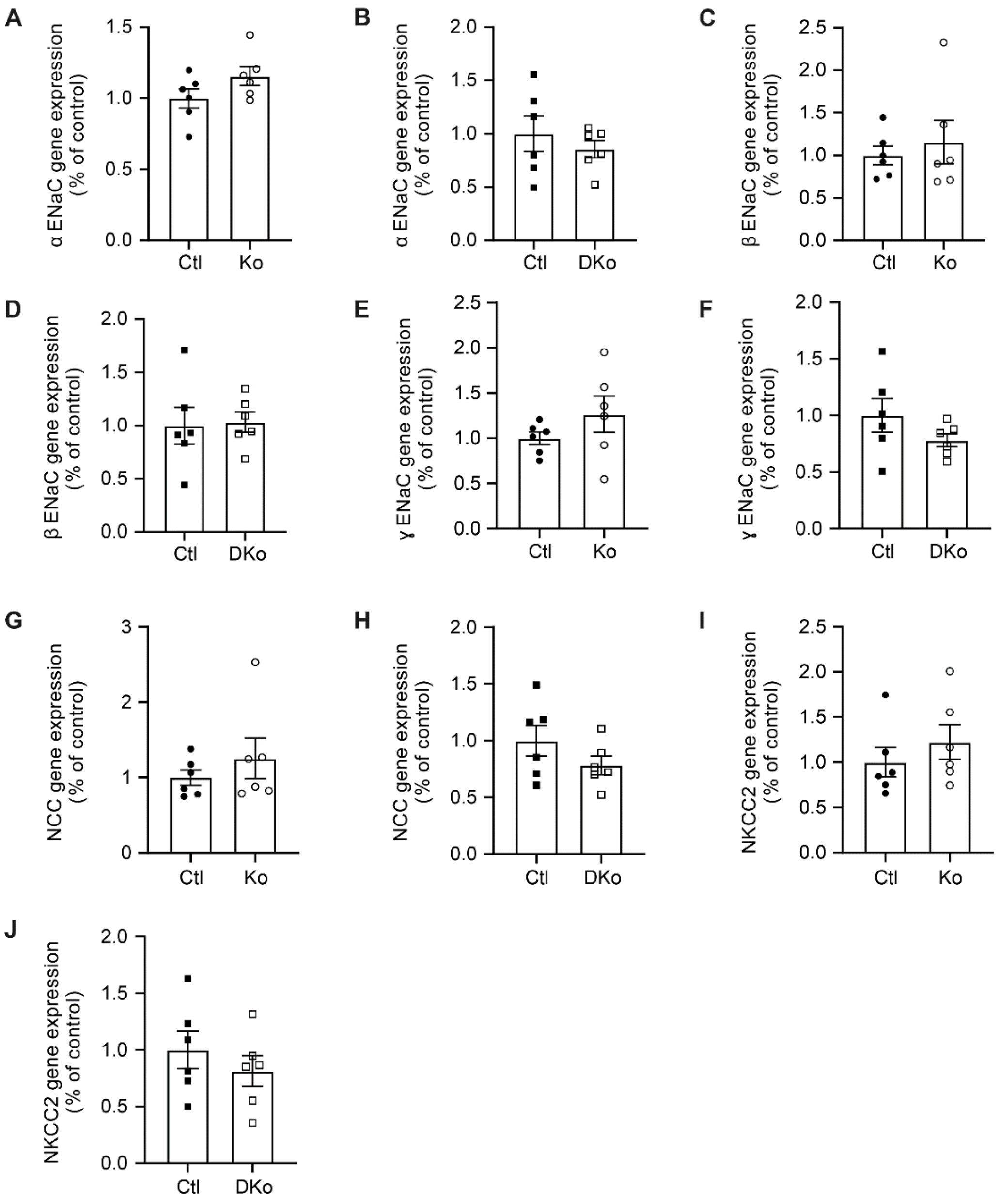

Figure S1. mRNA transcript expression of ENaC subunits do not differ in CAP3/St14 and CAP1/Prss8; CAP3/St14 double knockout mice. mRNA levels of **(A-B)** αENaC, **(C-D)** βENAC, **(E-F)** γENaC, **(G-H)** NCC, **(I-J)** NKCC2 in CAP3/St14 control (black circles, n=6) and CAP3/St14 knockout (white circle, n=6) mice and CAP1/Prss8; CAP3/St14 control (black square, n=6) and CAP1/Prss8; CAP3/St14 double knockout mice (white square, n=6) assessed by real-time PCR. Results are presented as mean ± SEM. Data were analyzed using an unpaired two-tailed Welch's t-test and p values<0.05 were considered as statistically significant; *p<0.05, **p<0.01.

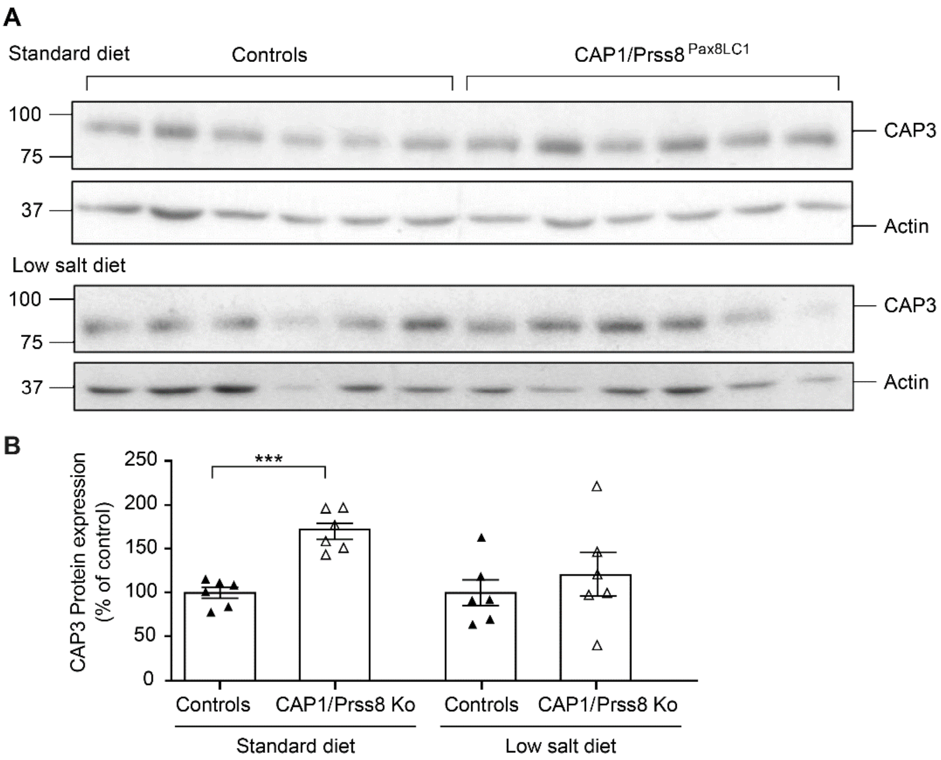

Figure S2. CAP3/St14 protein expression is increased in CAP1/Prss8 kidney-specific knockout mice under a standard Na^+ diet. **(A)** Representative Western blot analyses of CAP3 on kidney lysate from CAP1/Prss8 control (black triangle, n=6) and knockout mice (white triangle, n=6) under standard (up) and low Na^+ (down) diet. **(B)** Quantification of data under standard (left) and low salt (right) diet. Results are presented as mean ± SEM. Data were analyzed using an unpaired two-tailed Welch's t-test and p values <0.05 were considered as statistically significant; ***p<0.001.

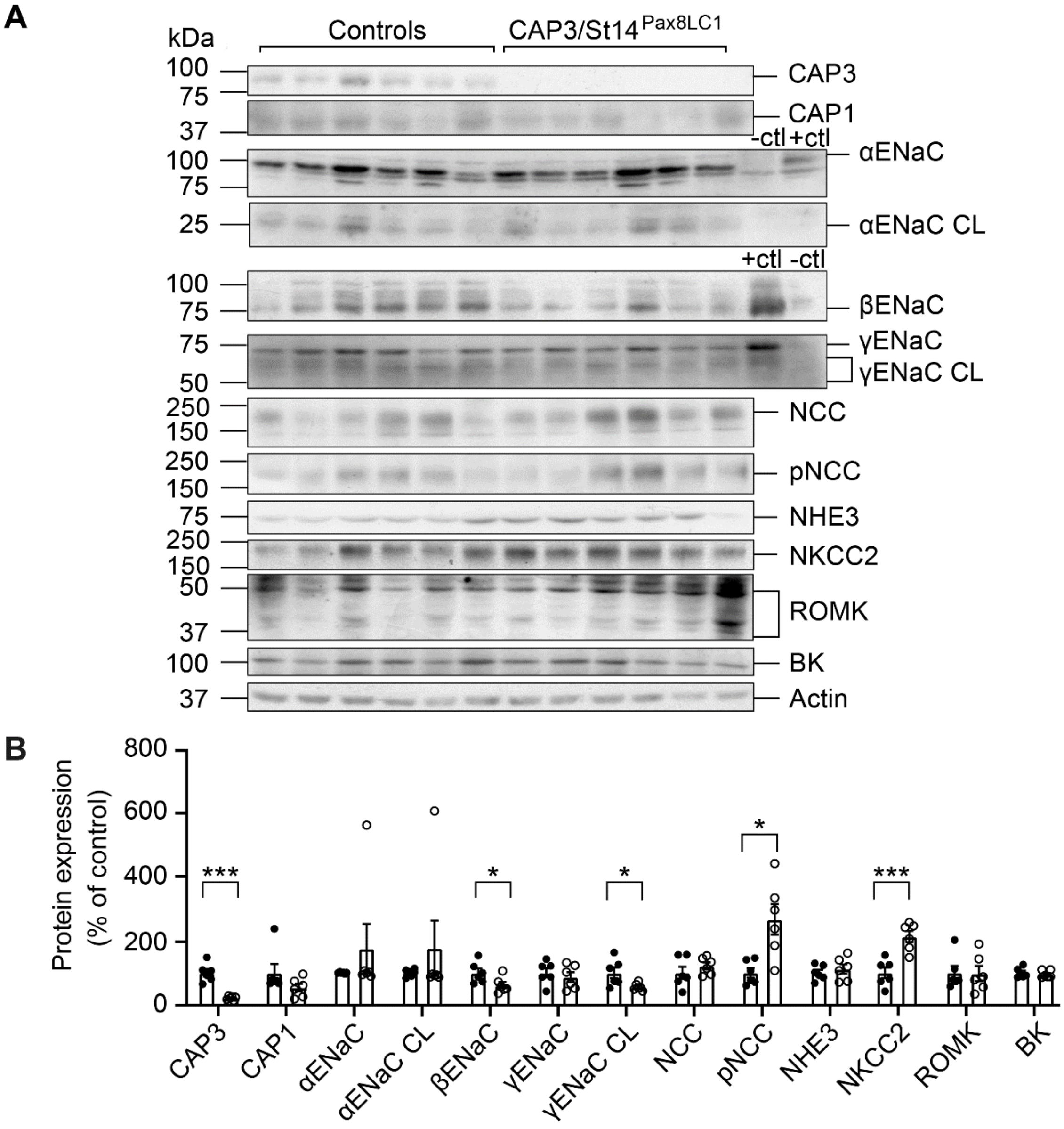

Figure S3. Protein expression of β- and γENaC cleaved form was decreased in CAP3/St14 knockout mice, whereas pNCC and NKCC2 were increased under a standard Na^+ diet. (**A**) Representative Western blot analysis of CAP3, CAP1, αENaC, αENaC CL (cleaved), βENaC, γENaC, γENaC CL (cleaved), NCC, pNCC, NHE3, NKCC2, ROMK and BK on kidney lysates from controls (black circles, n=6) and CAP3/St14 knockout (CAP3/St14^{Pax8LC1}, white circles, n=6) mice. Kidney lysates of control and renal tubular-specific knockouts of αENaC, βENaC, and γENaC served as positive (+ctl) and negative (-ctl) controls. (**B**) Quantification of the data. Results are presented as mean ± SEM. Data were analyzed using an unpaired two-tailed Welch's t-test.

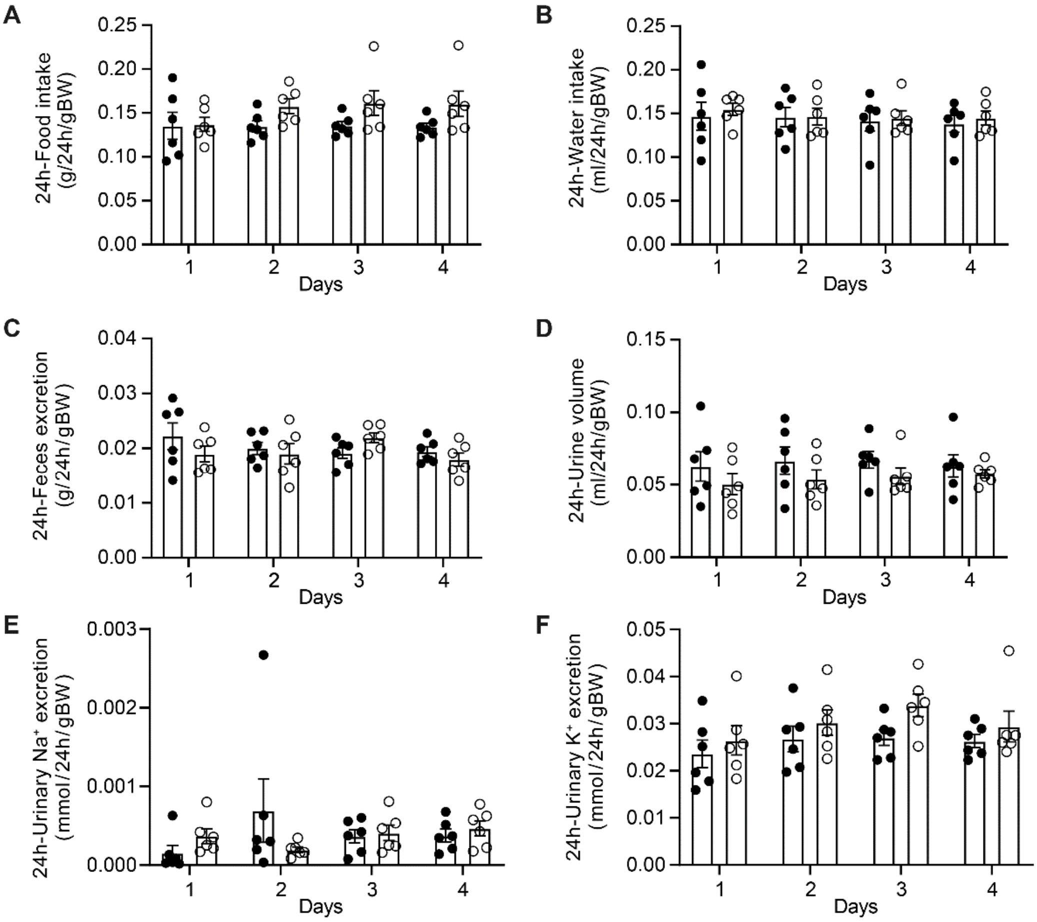

Figure S4. CAP3/St14 knockout mice displayed normal metabolic parameters under a low Na^+ diet. **(A)** Food (g/24h/gBW) and **(B)** water intake (ml/24h/gBW). **(C)** Feces excretion (g/24H/gBW), **(D)** Urine volume (ml/24h/gBW), **(E)** urinary Na^+ and **(F)** K^+ excretion (mmol/24h/gBW) in control (black circle, n=6) and CAP3/St14 knockout (white circles, n=6) mice. Results are presented as mean ± SEM. Data were analyzed by a two-way ANOVA with post hoc Šìdák multiple comparison test.

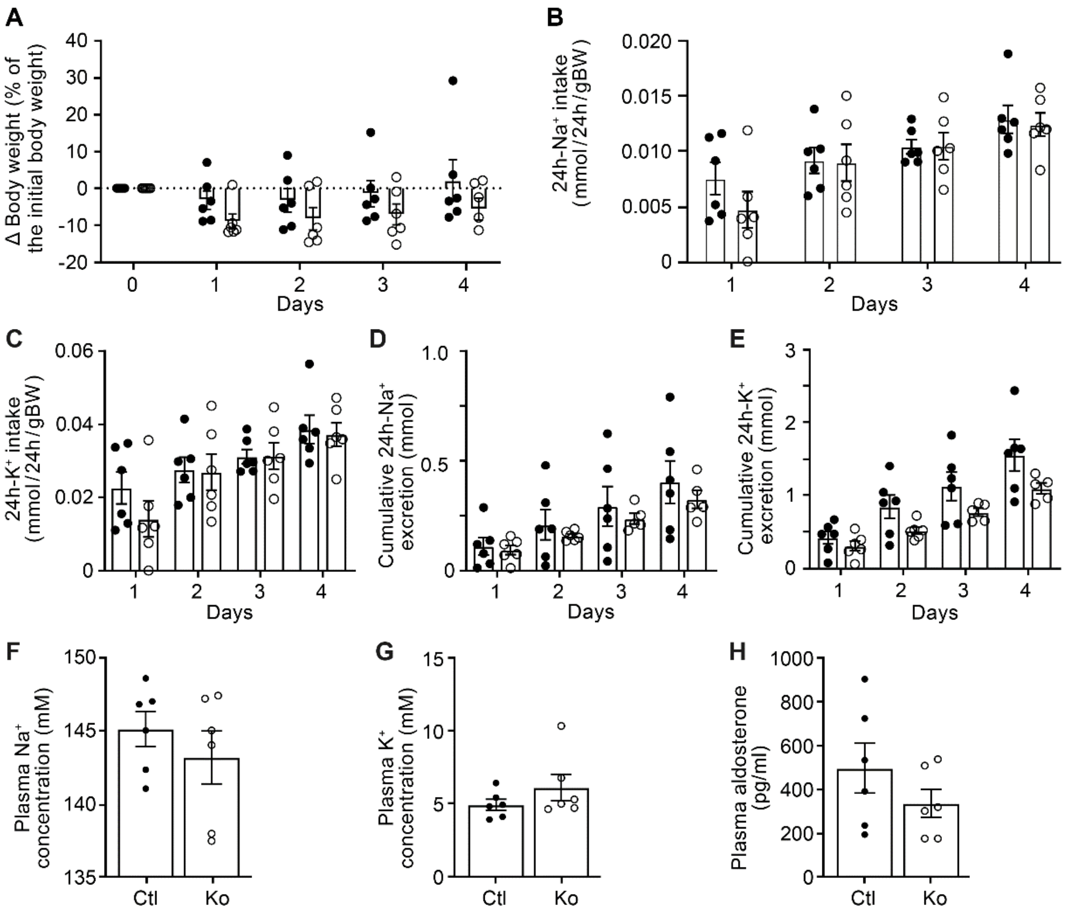

Figure S5: CAP3/St14 knockout mice displayed normal Na^+ and K^+ handling under a standard Na^+ diet**.** **(A)** Body weight changes (expressed as percent of initial body weight). **(B)** 24h Na^+ and **(C)** K^+ intake (mmol/24h/gBW) **(D)** 24h cumulative Na^+ and **(E)** K^+ excretion (mmol). **(F**) Plasma Na^+, **(G)** K^+ (mM) and **(H**) aldosterone concentration (pg/ml) in control (Ctl, black circles, n=6) and CAP3 knockout (Ko, white circles, n=6). Results are presented as mean ± SEM. **(A-E)** were analyzed by a two-way ANOVA with post hoc Šidák multiple comparison test. **(F-H)** were analyzed by an unpaired two-tailed Welch's t-test.

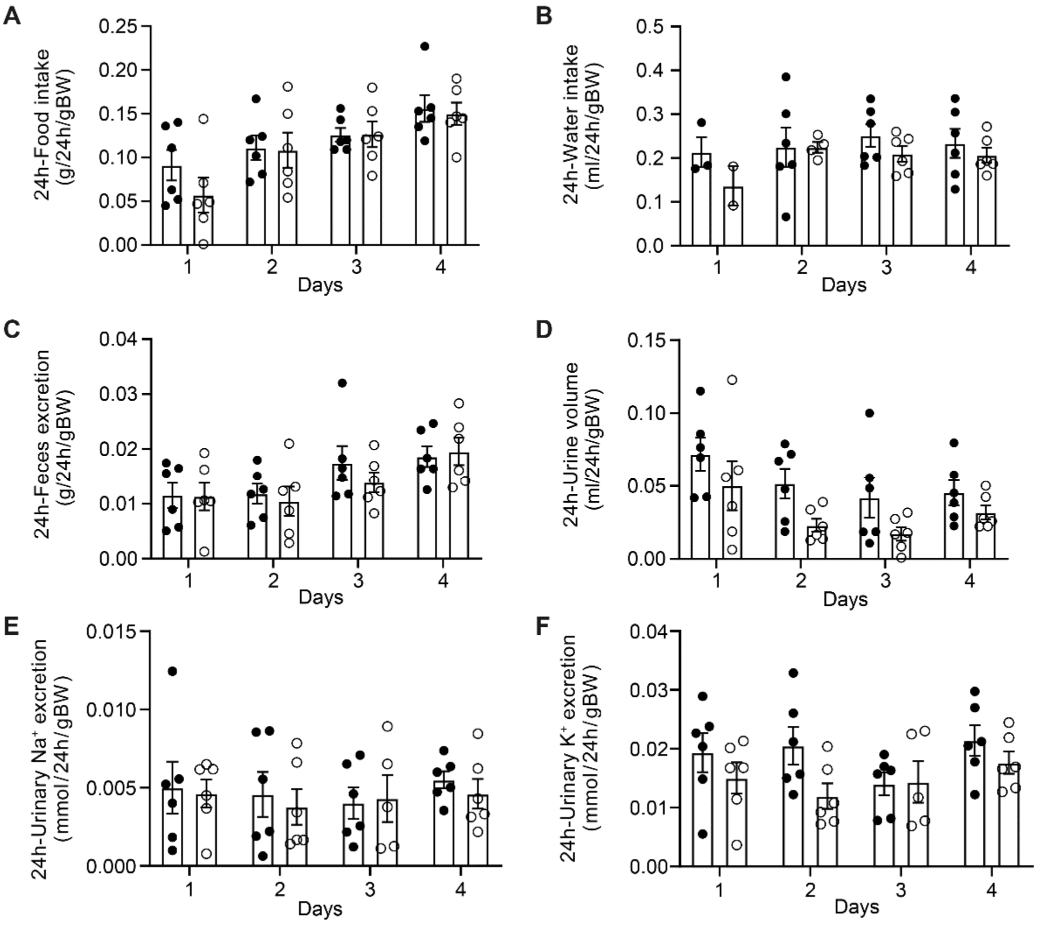

Figure S6. CAP3/St14 knockout mice displayed normal metabolic parameters under a standard Na^+ diet. **(A)** Food (g/24h/gBW) and **(B)** water intake (ml/24h/gBW). **(C)** Feces excretion (g/24H/gBW), **(D)** Urine volume (ml/24h/gBW), **(E)** urinary Na^+ and **(F)** K^+ excretion (mmol/24h/gBW) in control (black circle, n=3-6) and CAP3/St14 knockout (white circles, n=2-6) mice. Results are presented as mean ± SEM. Data were analyzed by a two-way ANOVA with post hoc Šìdák multiple comparison test.

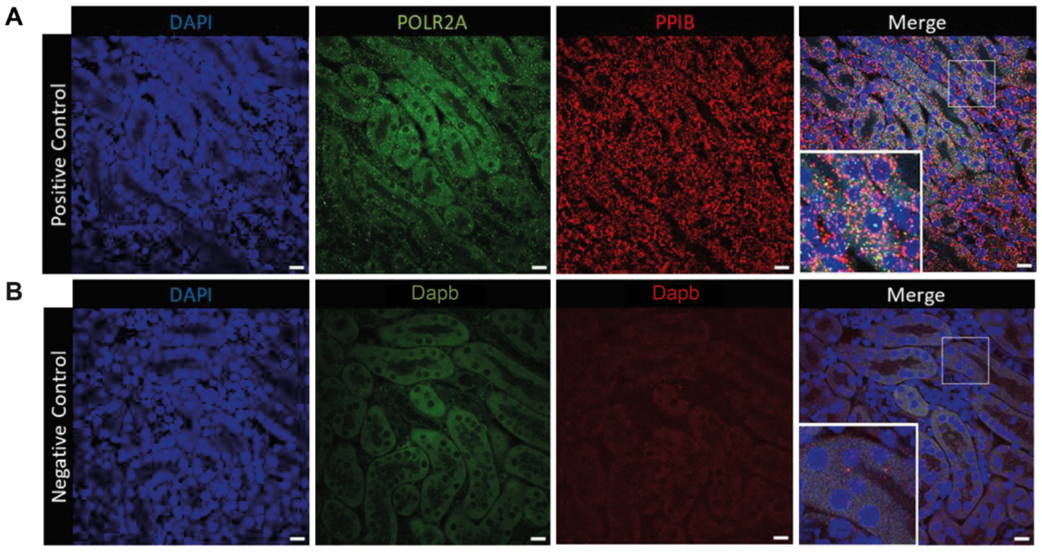

Figure S7: Positive and negative controls for St14 (CAP3), Scnn1a (αENaC) and Prss8 (CAP1) and fluorescent channel detection in kidney cortex from wildtype mice under a standard Na^+ diet. (**A**) Visualization of nuclei (DAPI staining, left) and expression of positive controls POLR2A (green middle left) and PPIB (red middle right), and merged picture (right). (**B**) Visualization of nuclei (DAPI staining, left) and expression of negative controls Dapb for green (middle left) and red (middle right) channels, and merged pictures (right). Magnification 40x, Scale bar represents 20µm.

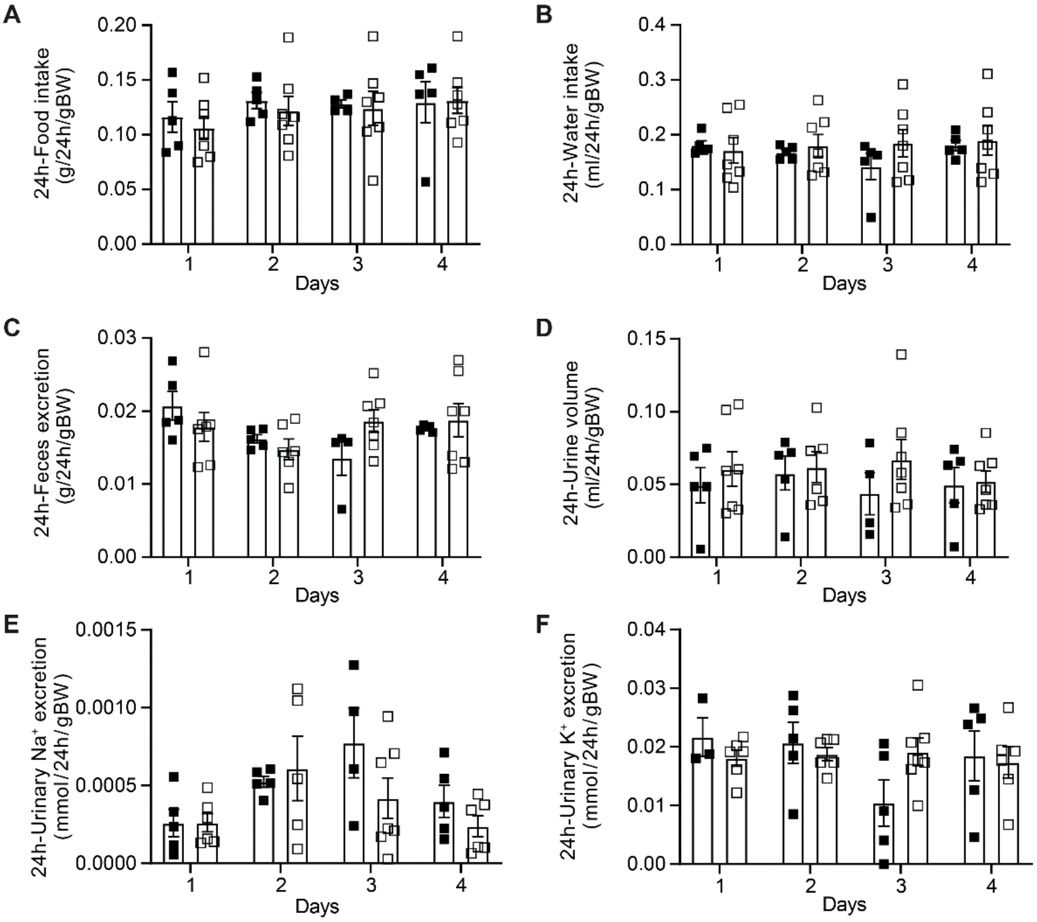

Figure S8. CAP1/Prss8; CAP3/St14 double knockout mice displayed normal metabolic parameters under a low Na^+ diet. **(A)** Food (g/24h/gBW) and **(B)** water intake (ml/24h/gBW). **(C)** Feces excretion (g/24H/gBW), **(D)** Urine volume (ml/24h/gBW), **(E)** urinary Na^+ and **(F)** K^+ excretion (mmol/24h/gBW) in control (black square, n=3-5) and CAP1/Prss8; CAP3/St14 double knockout (white square, n=5-7) mice. Results are presented as mean ± SEM. Data were analyzed by a two-way ANOVA with post hoc Šìdák multiple comparison test.

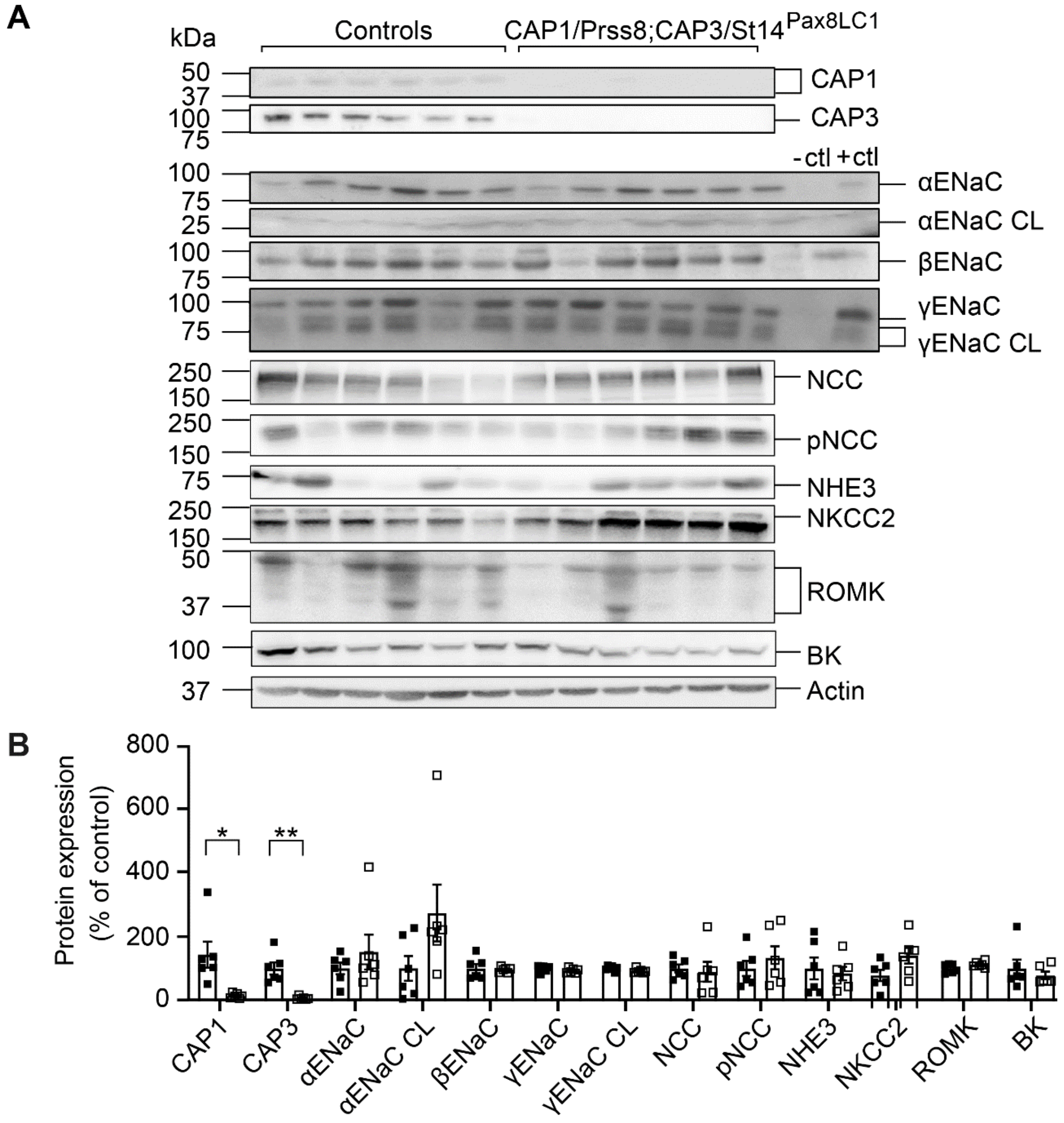

Figure S9: Normal abundance of Na^+ and K^+ transporting proteins in CAP1/Prss8; CAP3/St14 double knockout mice on a standard Na^+ diet. **(A)** Representative Western blot analysis of CAP1, CAP3, αENaC, αENaC CL (cleaved), βENaC, γENaC, γENaC CL (cleaved), NCC, pNCC, NHE3, NKCC2, ROMK and BK on kidney lysates from control (black squares, n=6) and CAP1/Prss8; CAP3/St14 double knockout (CAP1/Prss8;CAP3/St14^{Pax8LC1}, white spares, n=6) mice. Kidney lysates of control and renal tubular-specific knockouts of αENaC, βENaC, and γENaC served as positive (+ctl) and negative (-ctl) controls, respectively. **(B)** Quantification of the data. Results are presented as mean ± SEM. Data were analyzed by using an unpaired two-tailed Welch's t-test. P values <0.05 were considered statistically significant; *p<0.05, **p<0.01

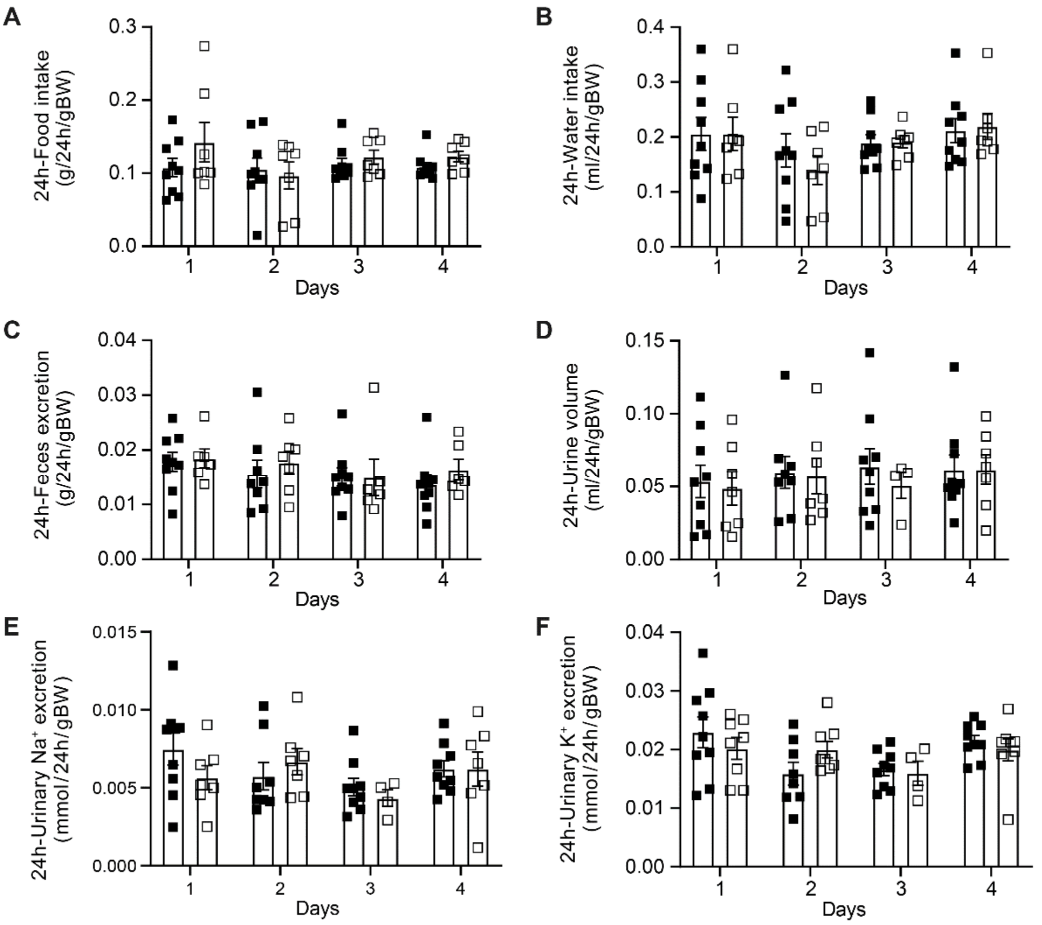

Figure S10: CAP1/Prss8; CAP3/St14 double knockout mice displayed normal metabolic parameters under a standard Na^+ diet. **(A)** Food (g/24h/gBW) and (**B)** water intake (ml/24h/gBW). **(C)** Feces excretion (g/24H/gBW), **(D)** Urine volume (ml/24h/gBW), **(E)** urinary Na^+ and **(F)** K^+ excretion (mmol/24h/gBW) in control (black square, n=9) and CAP1/Prss8; CAP3/St14 double knockout (white square, n=4-7) mice. Results are presented as mean ± SEM. Data were analyzed by a two-way ANOVA with post hoc Šìdák multiple comparison test.

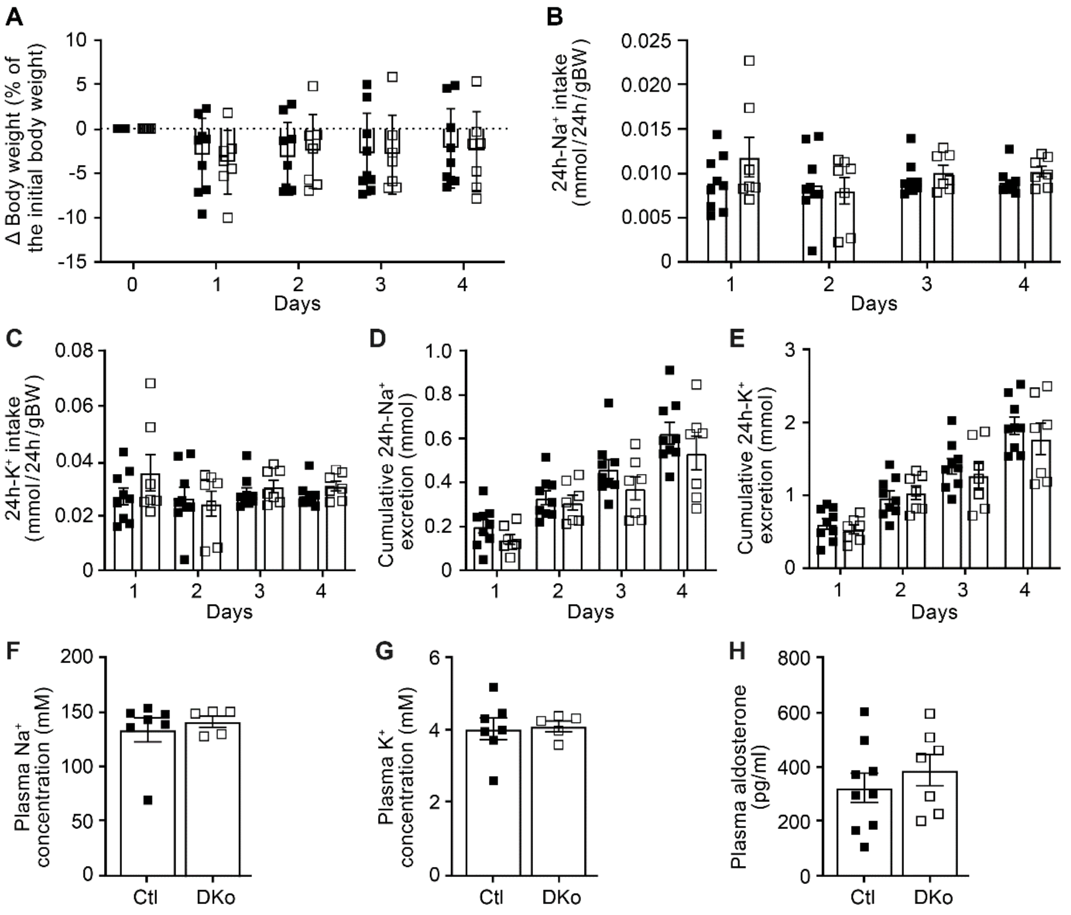

Figure S11: CAP1/Prss8; CAP3/St14 double knockout mice display normal Na^+ and K^+ handling under standard Na^+ diet. **(A)** Body weight changes (expressed as percent of initial body weight**)** **(B)** 24h Na^+ and **(C)** K^+ intake (mmol/24h/gBW**)**. **(D)** 24h cumulative Na^+ and **(E)** K^+ excretion (mmol). **(F)** Plasma Na^+, **(G)** K^+ (expressed in mM) and **(H)** aldosterone concentration (pg/ml) in control (Ctl, black squares, n=7-9) and CAP1/Prss8; CAP3/St14 double knockout (DKo, white squares, n=5-7). Results are presented as mean ± SEM**. (A-E)** were analyzed by a two-way ANOVA with post hoc Šìdák multiple comparison test**. (F-H)** were analyzed by an unpaired two-tailed Welch's t-test.

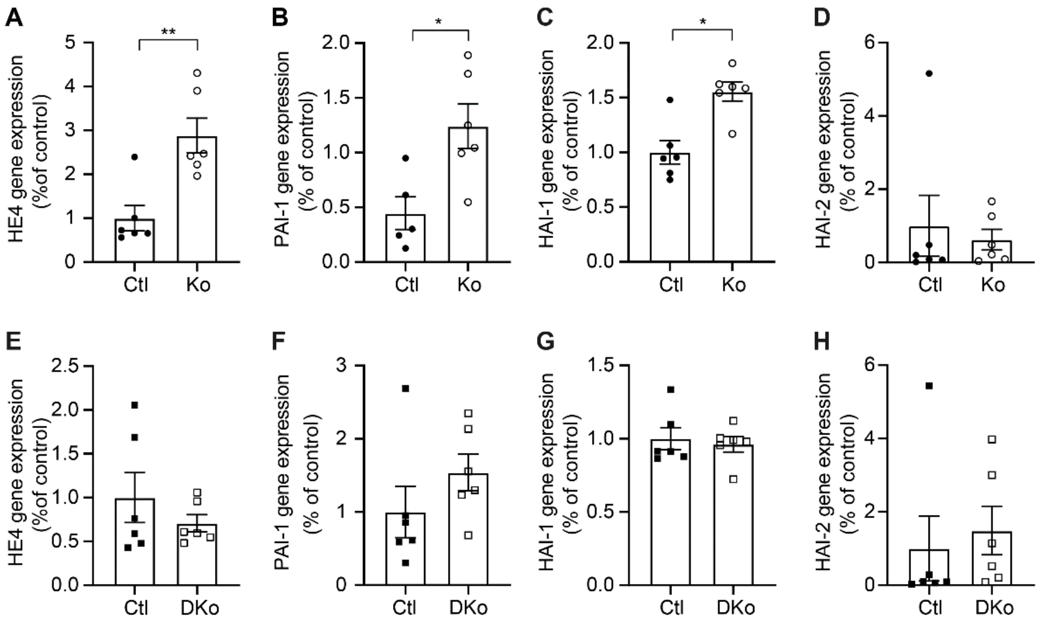

Figure S12: mRNA transcript expression of HE4, PAI-1 and HAI-1 is increased in CAP3/S14 knockout mice under low Na^+ diet. mRNA levels of (A,E) HE4, (B,F) PAI-1, (C,G) HAI-1, (D,H) HAI-2 in (A-D) CAP3/St14 control (black circles, n=6) and CAP3/St14 knockout (white circle, n=6) mice and (E-H) CAP1/Prss8; CAP3/St14 control (black square, n=6) and CAP1/Prss8; CAP3/St14 double knockout mice (white square, n=6) assessed by real-time PCR. Results are presented as mean ± SEM. Data were analyzed using an unpaired two-tailed Welch's t-test and p values<0.05 were considered as statistically significant; *p<0.05, **p<0.01.
